# Evaluating the Public Climate School—A School-Based Programme to Promote Climate Awareness and Action in Students: Protocol of a Cluster-Controlled Pilot Study

**DOI:** 10.3390/ijerph19138039

**Published:** 2022-06-30

**Authors:** Michael Eichinger, Myriam Bechtoldt, Inga Thao My Bui, Julius Grund, Jan Keller, Ashley G. Lau, Shuyan Liu, Michael Neuber, Felix Peter, Carina Pohle, Gerhard Reese, Fabian Schäfer, Stephan Heinzel

**Affiliations:** 1 Center for Preventive Medicine and Digital Health, Medical Faculty Mannheim, Heidelberg University, 68167 Mannheim, Germany; 2 Division of Pediatric Epidemiology, Institute of Medical Biostatistics, Epidemiology and Informatics, University Medical Center of the Johannes Gutenberg, University of Mainz, 55131 Mainz, Germany; 3 Department of Management, EBS University of Business and Law, 65375 Oestrich-Winkel, Germany; myriam.bechtoldt@ebs.edu; 4 Klimabildung e.V., 44787 Bochum, Germany; thaomybui@googlemail.com (I.T.M.B.); carina.pohle@posteo.de (C.P.); fabian@klimabildung.org (F.S.); 5 Institut Futur, Freie Universität Berlin, 14195 Berlin, Germany; grund@institutfutur.de; 6 Department of Education and Psychology, Freie Universität Berlin, 14195 Berlin, Germany; jan.keller@fu-berlin.de (J.K.); heinzel@zedat.fu-berlin.de (S.H.); 7 Department of Psychiatry and Psychotherapy, Campus Charité Mitte, Charité—Universitätsmedizin Berlin, 10117 Berlin, Germany; ashley.lau@charite.de (A.G.L.); or shuyan.liu@charite.de (S.L.); 8 Center for Technology and Society, Technical University of Berlin, 10553 Berlin, Germany; mineuber@uni-potsdam.de; 9 Psychologists for Future e.V., 55411 Bingen, Germany; felix.peter@posteo.de; 10 Department of Social, Environmental and Economic Psychology, Faculty of Psychology, University of Koblenz Landau, 76829 Landau, Germany; reese@uni-landau.de; 11 Center for Economic Education, Department of Economics, Ruhr University Bochum, 44801 Bochum, Germany

**Keywords:** education for sustainable development, climate change, planetary health, self-efficacy, collective efficacy, risk perception, behavioural intentions, pro-environmental behaviour, climate anxiety

## Abstract

**Introduction:** School-based programmes may promote knowledge and skills required to address climate change and better health and well-being in adolescents, yet evidence of their effectiveness is limited. In preparation for evaluating the Public Climate School, a school-based intervention to promote climate awareness and action in adolescents, we conduct a pilot study intended to assess procedures for participant recruitment, retention, and data collection, data quality issues and to provide preliminary parameter estimates to guide sample size calculations. **Methods and analysis:** This unblinded, cluster-controlled pilot study targets students in twelve classes from grades seven to thirteen in German public schools. Seven and five classes were allocated to the intervention and waitlist control arms, respectively. The intervention consisted of (1) live lessons on YouTube, (2) climate-related challenges of the day, (3) workshops and (4) peer exchange sessions. Waitlist control classes participated three weeks later. Measures included the proportion of students completing baseline and follow-up surveys, a comparison of baseline characteristics between students in the retained subsample and those lost to follow-up, proportions of students completing online and paper–pencil-based surveys and problems during data collection based on information reported by teachers. Data quality was assessed as proportions of missing data, associations between missingness and sociodemographic measures using logistic regression models and basic psychometric properties of scales including ceiling effects and internal consistency. Intentions to reduce one’s ecological footprint, the primary outcome, and all secondary outcomes for effect estimation were assessed one week pre- and post-intervention from November to December 2021 using items adapted from internationally used instruments and will be investigated using generalised linear mixed models and intention-to-treat analyses. **Conclusions:** The pilot study will lay the methodological groundwork for a large-scale cluster-randomised effectiveness and process evaluation of the Public Climate School. If proven effective and rolled out more broadly, the Public Climate School has the potential to contribute meaningfully to national climate mitigation and adaptation efforts by reaching a substantial share of adolescents in public schools, including those traditionally less involved in climate action.

## 1. Introduction

Anthropogenic climate change is the biggest threat to health in the 21st century and has the potential to disrupt planetary systems as we know them [1,2,3,4,5,6,7]. Adolescents are particularly vulnerable to the negative effects of the accelerating climate crisis [8,9], particularly in terms of negative mental health effects [10,11,12]. Anxiety concerning the consequences of climate change is widespread among adolescents and impacts their daily functioning [13]. Moreover, a recent review highlighted that climate change places adolescents at risk of further adverse mental health outcomes, including post-traumatic stress disorder, depression, sleep disorders and substance abuse [10].

Adolescents can, however, play an important role as drivers of change. Together with other civil society stakeholders, they have propelled the Fridays for Future movement, demanding ambitious action to tackle the climate crisis at national and international levels [14], and contribute to numerous sustainability initiatives locally [15]. Additionally, preliminary evidence suggests a positive association between sustainability-related activities and individual well-being, pointing to the potential of adolescents’ climate action for the prevention of climate-associated mental health problems [16]. These observations provide support for ongoing efforts to promote climate mitigation and adaptation activities among adolescents.

To safeguard acceptable living conditions for future generations, approaches are needed that actively engage a broad spectrum of citizens in climate change mitigation and adaptation. In this context, large-scale school-based programmes may be useful as school attendance is mandatory in most countries, and programmes promoting climate awareness and action in schools could thus reach a substantial proportion of young people. School-based programmes have the potential to increase knowledge about the climate crisis, empower students to become change agents and thus provide a basis for them to initiate and contribute to meaningful climate action [17,18]. Recent evidence also suggests that both students and teachers desire substantially more opportunities for education on sustainable development (ESD) [19]. However, despite its greater implementation within school curricula, current efforts have fallen short in Germany and elsewhere [20,21].

Despite the potential of school-based programmes, empirical evidence on their effectiveness remains limited. Results of observational studies remain largely inconsistent, with some showing positive associations between ESD and students’ sustainability knowledge [22,23], attitudes [22,24] and behaviours [25] and others showing no relationship for the same outcomes [25]. To our knowledge, studies that test the effects of school-based ESD interventions on climate awareness and action, explore mechanisms linking these interventions to intended outcomes and assess contextual factors that potentially moderate intervention effectiveness, do not exist [19].

Recently, the Public Climate School (PCS), a multi-component school-based programme aiming to promote climate awareness and action among school students, has been developed and offered in collaboration with public schools in Germany to approximately 30,000 students each year since 2019 by Students for Future, an offshoot of the Fridays for Future movement. The content and delivery of the PCS is guided by the concept of ESD [26] and focuses on, inter alia, participatory teaching and learning methods, critical thinking and imagining future scenarios [27]. The PCS is a complex intervention comprising several interacting components, such as live online lessons, interactive workshops and peer exchange sessions, and engages different stakeholder groups including students, teachers, school principals and the team implementing the PCS. Anticipated effects, potentially influenced by a variety of contextual factors, include increased individual and collective efficacy beliefs, increased identification with civil society groups involved in climate action and pro-environmental behavioural intentions of students. To date, however, an evaluation of this programme has not been undertaken.

In the proposed EvalPCS Pilot Study, Students for Future partner with academics spanning diverse disciplines, including psychology, epidemiology, education research and sociology, to address feasibility concerns related to participant recruitment and retention, data collection, data quality and the required sample size for a future cluster-randomised effectiveness study. The pilot study thereby aims to lay the groundwork for a future comprehensive evaluation of PCS programme effectiveness, mechanisms of action and contextual factors affecting its implementation and effectiveness.

### Objectives

In this article, we describe the design of the EvalPCS Pilot Study. As primary objectives, we investigate:

(1) Participant recruitment and retention;
(2) Data collection;
(3) Data quality.

As a secondary objective, we will obtain preliminary parameter estimates contributing to future sample size calculations. 

## 2. Methods and Analysis

### 2.1. Study Design

**Figure 1:**
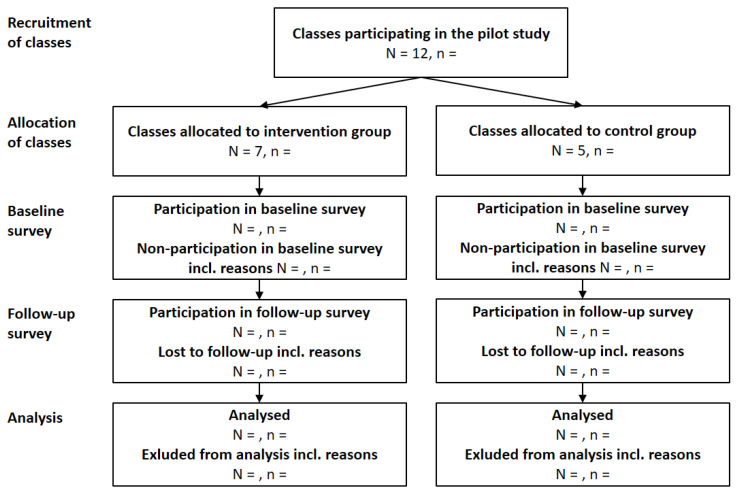
**CONSORT flowchart.** N, number of schools; n, number of adolescents.

The study design reflects the updated UK Medical Research Council guidance on the development and evaluation of complex interventions and focuses on investigating the feasibility of the evaluation design, one of four phases in complex intervention research [28]. The pilot study is based on a two-arm, cluster-controlled design assessing the feasibility of the PCS evaluation design in five schools with seven classes receiving the PCS (henceforth intervention classes) and five classes serving as waitlist controls that received the PCS three weeks later (henceforth control classes; Figure 1).

### 2.2. Study Status

Allocation to the intervention and control groups was completed by November 19, 2021, and the PCS was implemented between 22 and 26 November 2021. Data collection was concluded by December 2021. Data cleaning and analysis is anticipated to start in May 2022.

### 2.3. Setting

All schools in Germany, without restrictions in terms of location, school type and grade, can participate in the PCS. To invite schools, we disseminated information on the content and delivery of the PCS via all state ministries of education. In addition, we used social media channels and newsletters of the Fridays for Future, Students for Future and Teachers for Future networks, the PCS newsletter and the network of schools established during previous offerings of the PCS in November 2020 and May 2021.

### 2.4. Recruitment Strategy

An overview of the recruitment strategy is provided in the CONSORT flowchart (Figure 1). While all classes of any grade including primary schools can participate in the PCS, the pilot study specifically targeted adolescents. We therefore recruited a convenience sample of classes covering grades 7 to 13 (mostly attended by adolescents aged 12 to 19 years) to participate in the pilot study. With the aim of preparing a pragmatic effectiveness study, we did not specify further inclusion or exclusion criteria for classes. In October 2021, using the newsletter of the PCS, we invited all 7500 schools in 14 federal states as well as 64 schools that had participated in previous PCS and were registered for the present PCS to participate in the pilot study. By 19 November 2021, twelve classes from five schools had consented to participate in the pilot study. 

To recruit adolescents, we invited all students in participating classes to take part in the pilot study by distributing a leaflet on the purpose and conduct of the study. All student participants and their parents provided written informed consent. To strengthen the generalisability of our findings, we did not apply any inclusion or exclusion criteria. However, students lacking German language skills that would allow them to complete a short questionnaire in German were excluded de facto.

### 2.5. Intervention

The goal of the PCS is to increase students’ knowledge about the climate crisis, empower them to become change agents and thus to create the basis for initiating and helping to shape far-reaching climate action. With the aim of bridging the gap between knowledge and action, the PCS goes beyond pure knowledge transfer and focuses more on action-oriented learning in projects, role plays and encounters.

Together with experts from science and society, the implementation team developed the PCS in 2019. Although initially targeted at university students, the PCS was first implemented in November 2020 in primary and secondary schools using tailored content and materials. The PCS is rooted in ESD with its principles of forward thinking, interdisciplinary knowledge, autonomous action and participation in social decision-making processes [29]. Moreover, the PCS is guided by the 17 United Nations Sustainable Development Goals. Prior to its first implementation in November 2020, one lesson was pretested with one class to test technical and on-site procedures at the school. No changes were made to content or delivery after the pretest. Since this time, the content and delivery of the PCS has been constantly refined based on field reports and reflection meetings of the implementation team. 

In its current form, the PCS lasts for five days, is implemented twice a year in May and November and focuses on the climate crisis and related topics such as biodiversity loss [30], climate justice (i.e., attitudes and actions supporting the just division and fair distribution of benefits and burdens of and responsibilities to tackle climate change) [31], climate communication [1], economic impact of the climate crisis [32], visions of a sustainable future [33] or climate and arts [34]. Based on prior work suggesting positive effects of tailoring strategies on intervention effectiveness [35], the PCS is tailored to local needs at two levels. At the level of classes, teachers decide autonomously which intervention components they take part in. At the level of intervention components, complementing the highly standardised live lessons delivered centrally by the implementation team, the challenges of the day, workshops and peer exchange sessions are tailored to local students’ needs. 

Intervention components developed and implemented to this point include:

**(1) Live lessons on YouTube:** Four standalone live lessons per day that can be incorporated into classroom teaching individually are the backbone of the PCS. Lessons last 45 min each and are broadcast via the PCS YouTube channel. The lessons are suitable for different grades and are delivered by teams of students with activist backgrounds and subject experts, including university teachers and researchers, from different institutions with expertise in the focus area of the respective lesson. The lessons, which emphasise the importance of critical thinking, provide basic knowledge about the climate crisis and its impact in different social and scientific fields (e.g., economics, politics). 

Students follow live broadcasts on YouTube and are encouraged to actively participate in lessons. Active participation is encouraged by several aspects. First, in addition to phases focused on building knowledge, each lesson comprises a phase for discussions and mutual exchange between students. Second, survey tools enable online interaction between experts and students. Third, worksheets accompanying each lesson invite students to become active themselves.

During the PCS, the implementation team collaborates with teachers on site. While the presenters are primarily responsible for the actual knowledge transfer, the teachers are in direct contact with students and provide technical support and non-technical assistance, including the distribution of worksheets. 

Live lessons are recorded and provided online together with worksheets and other supplementary materials, including syllabi and podcasts (https://publicclimateschool.de, accessed on 1 March 2022). This enables asynchronous participation in the PCS and makes participation of teachers outside their subject area possible.

**(2) Climate-related challenges of the day:** On each day of the PCS, classes can optionally participate in the challenge of the day, a format that encourages students to further explore specific topics and enables them to take action by initiating their own projects and activities. Activities include, inter alia, recording impacts of humans on the environment in poems, podcasts or videos, preparation of material for climate protests, writing letters to politicians at local, state or federal levels demanding comprehensive mitigation and adaptation measures or identifying further possibilities to take action on the climate crisis. The challenges aim at strengthening students’ self-efficacy and agency. They are announced directly before the live sessions on the YouTube channel and are published on the PCS website. Participating teachers and students decide autonomously whether and how to participate in the challenges. Reporting of activities to the implementation team is at the discretion of teachers.

**(3) Workshops:** Workshops are interactive and focus on specific topics such as food waste, climate justice, climate-induced migration or creative writing in light of the climate crisis. The workshops are delivered online or on site at schools by cooperation partners such as other educational institutions. The implementation team discusses the workshops with the organisers in advance but is not involved in content development or delivery.

**(4) Peer exchange sessions:** Peer exchange sessions include presentations of good practice examples and provide opportunities for participants to share their own experiences and projects with each other and to network. Peer exchange sessions are facilitated by members of the implementation team.

Teachers and school principals interested in participating in the PCS receive detailed information about the aims and procedures of the PCS during online events run by the implementation team prior to the start of the PCS. 

### 2.6. Social Identity Model of Pro-Environmental Action as Tentative Programme Theory

**Figure 2:**
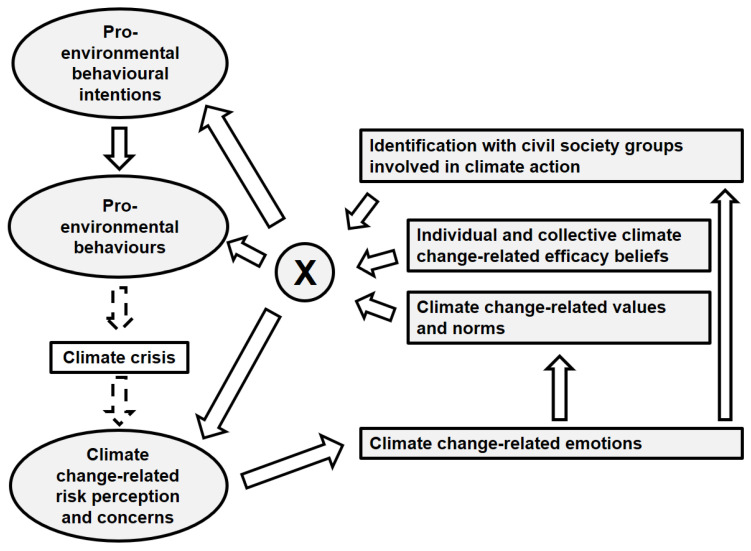
**The Social Identity Model of Pro-Environmental Action (SIMPEA) as a tentative programme theory of the Public Climate School.** Arrows indicate proposed causal pathways between components of the model. The “X” indicates the anticipated interaction between social identity-based factors including identification with pro-environmental in-groups, efficacy beliefs and salient pro-environmental values and norms.

Previous work highlights the value of applying a programme theory a priori in the development and evaluation of complex interventions [28]. While several concepts, such as the principles of ESD, guided the development, content and delivery of the PCS, in the absence of a previously established programme theory, we used the Social Identity Model of Pro-Environmental Action (SIMPEA) [36] to guide the selection of outcomes for this pilot study (Figure 2). While there are various other theoretical models in the field that could explain climate action, including the theory of planned behaviour, the norm activation theory and the comprehensive action determination model [37,38,39], we chose the SIMPEA because, unlike the other models, it specifically intends to explain the complex interplay of collective factors that influence the appraisal of environmental crises and thus climate action, the main objective of the PCS. 

According to the model, pro-environmental behaviour is a response to the climate crisis aimed at attenuating human-induced climate change and its impacts. This response, in turn, is a function of social identity-based predictors: the more strongly people identify with a relevant pro-environmental in-group, the more efficacious they feel, and the more salient respective pro-environmental values and norms within a group are, the higher the likelihood of performing pro-environmental behaviours. Specifically, the model proposes that the interaction of these variables affects both the appraisal of environmental crises that, in turn, can trigger (collective) emotions that affect individuals’ efficacy beliefs, norms and whether and how people identify with certain groups, as well as responses.

This recursive model has recently been applied in the context of protests for climate justice [40], and it has become a useful framework for explaining social and psychological determinants of climate action (e.g., pro-environmental behaviour directed towards reducing greenhouse gas emissions) [41]. Applying this model to the PCS would therefore suggest effects on increased intentions to engage in climate action by, inter alia, (1) increasing identification with civil society groups involved in climate action, (2) affecting emotions related to the climate crisis and (3) improving individual and collective efficacy beliefs. 

### 2.7. Allocation of School Classes to Intervention or Control Group

The unit of allocation was school classes. Due to logistical constraints, allocation to the intervention and control group was performed locally by teachers. Teachers participating in the pilot study with two classes allocated the classes by tossing a coin. Teachers participating in the pilot study with one class only allocated their class to the intervention or control group without constraints.

### 2.8. Allocation Concealment and Blinding

Classes were allocated to intervention and control groups directly after recruitment ensuring allocation concealment. Due to the nature of the intervention, it is not possible to blind students, teachers, school principals or the implementation team to the intervention status of a school class, but researchers analysing the data will be blinded.

### 2.9. Measures

#### 2.9.1. Process Measures

We operationalised measures of study processes as follows:

- Participant recruitment and retention:
  ○ Proportion of students in participating classes completing the baseline survey;
  ○ Proportion of students in participating classes completing the follow-up survey;
  ○ Proportion of students in participating classes completing both baseline and follow-up surveys.
- Data collection:
  ○ Proportions of students participating in the online and paper–pencil surveys;
  ○ Problems during data collection.
- Data quality:
  ○ Proportion of missing data for outcome and sociodemographic measures;
  ○ Basic psychometric properties of scales used to assess programme effectiveness (Table 1) including scale distributions, floor and ceiling effects and internal consistency.

#### 2.9.2. Outcome Measures

Our selection of the primary and second outcomes was guided by the SIMPEA model (Table 1) [36]. Given that validated scales for assessing most outcomes were not available in German, we relied on items translated and adapted from international studies. When suitable items were not available, new items were created by the study team. The student survey is provided in a supplementary online appendix.

#### 2.9.3. Sociodemographic Measures

Sociodemographic data were assessed at baseline and included age, gender, parental educational attainment, municipal size and subjective social status using the German version of the MacArthur Scale [57].

### 2.10. Data Collection

Data were collected in both intervention and control classes one week before and one week after the PCS (Figure 1). Students accessed and completed an online survey (Unipark Version 21.2, QuestBack GmbH, Oslo, Norway) at both time points via a link/QR code provided in the invitation leaflet, using their private devices during school hours. A paper-and-pencil version with the same content was also offered. To minimise loss to follow-up, we sent two reminders to encourage participation. Teachers were invited to complete a teacher protocol sheet assessing the number of eligible students and problems during the evaluation process.

### 2.11. Sample Size Considerations

Recent methodological evidence suggests that feasibly sized pilot studies for cluster randomised trials typically lack precision in reliably estimating recruitment and retention rates and estimates of sample sizes are biased downwards [58]. Sample size considerations for the pilot study were thus geared towards requirements for investigating data collection and data quality for which no formal statistical guidance exists. We therefore pragmatically aimed to recruit 15 school classes with 10–15 students each, yielding a total sample of 150–225 consenting participants.

### 2.12. Analysis

To assess participant retention, we will examine differences between the retained subsample (data provided on both assessments) and the subsample lost to follow-up regarding baseline values of sociodemographic and outcome measures using a dichotomous retention variable and logistic regression models. Proportions of students participating in the online and paper–pencil survey modes will be analysed descriptively. Problems during data collection will be abstracted from reports submitted by teachers and structured thematically to improve the implementation of the future effectiveness and process evaluation.

To uncover missingness patterns [59], missing data will be assessed across different variables. To detect potential missingness mechanisms, we will investigate associations between dichotomous variables identifying respondents with missing data and sociodemographic measures using logistic regression models. Cronbach’s alpha will be used to investigate internal consistency of scales. After computing mean scores, scale distributions as well as floor and ceiling effects will be analysed descriptively.

In a randomisation check, we will examine the balance of outcomes and sociodemographic measures in the baseline data between the intervention and control arm. The continuous primary outcome (intentions of students to reduce their ecological footprint) will be compared between the intervention and control arm with an intention-to-treat analysis using a linear mixed model containing a dichotomous intervention group variable and a random effect for school classes to account for the nested data structure [60]. Secondary outcomes will be compared between the two study arms using analogous linear mixed models.

### 2.13. Reporting, Ethics and Study Registration

The reporting in this study protocol is based on the SPIRIT 2013 guideline for reporting protocols of intervention trials [61], the CONSORT extension for randomised pilot and feasibility trials [62] and guidance for the reporting of protocols of pilot and feasibility studies [63].

The study was approved by the Ethics Review Board of Freie Universität Berlin (036/2021) and school authorities of federal states in which classes participated in the pilot study. Written informed consent was obtained from students and their parents. Participation was voluntary, and no financial incentives were offered to either students or their teachers. The study was prospectively registered in the German Clinical Trials Register on 18 November 2021 (registration number: DRKS00027021).

## 3. Discussion

The proposed study will add to the scarce literature investigating school-based programmes to promote climate awareness and action among adolescents. Given that almost all adolescents aged under 16 years and approximately 87% of adolescents aged 16 to 19 attend schools on a regular basis in Germany [64], the PCS has the potential to reach a substantial share of adolescents. By involving broad groups of adolescents in the PCS, we anticipate that the school-based programme will contribute to extending proactive participation in climate change efforts beyond adolescents from higher socioeconomic backgrounds reported to be traditionally involved in climate action to a larger extent [65,66]. The PCS thus has the potential to contribute meaningfully to national climate mitigation and adaptation efforts if proven effective and rolled out more broadly.

In contrast to prior programme evaluations that often rushed into large-scale effectiveness studies, we will follow recent guidance on the development and evaluation of complex interventions and conduct a comprehensive feasibility study focusing on important uncertainties of the evaluation design [28]. The pilot study will thus lay solid groundwork for a comprehensive effectiveness and process evaluation including the investigation of implementation, mechanisms linking the PCS to its intended outcomes and contextual factors moderating implementation and intervention effectiveness. The primary objectives of the pilot study are to investigate participant recruitment and retention, data collection and data quality. The uptake of the online and paper–pencil survey modes will guide the selection of survey modes for the full-scale evaluation, while considering the trade-off between providing students low-threshold opportunities to participate in the study and minimising the burden for schools associated with administering multiple survey modes. The assessment of missing data will uncover potential to improve structure and content of the student surveys. The investigation of basic psychometric properties of the primary and secondary endpoints such as scale distributions and floor or ceiling effects will identify items and scales with poor psychometric performance and will provide opportunities to improve items and scales prior to the large-scale effectiveness evaluation.

As a secondary objective, we will obtain initial parameter estimates for the primary endpoint of the PCS along with recruitment and retention rates to better guide sample size estimation for the planned effectiveness study. As estimates of sample size based on feasibly sized pilot studies for cluster-randomised studies tend to be biased downwards [58], we will combine results of the pilot study with external evidence including published lists of intra-class correlation coefficients (ICC) and general considerations on patterns of ICCs, to guide estimates for sample size and recruitment and retention rates for the main trial, as suggested in the literature [67]. Preliminary estimates of parameters and ICCs from the pilot study may themselves feed into sample size estimations of similar school-based programmes in Germany and internationally. 

In summary, the proposed pilot study will provide a solid groundwork for a large-scale cluster-randomised effectiveness and process evaluation of the PCS. The pilot study therefore will better position future efforts to develop best-practice models for promoting climate awareness and action in adolescents and comparable educational programmes in other grades, school types or countries.

### Limitations

We acknowledge several limitations of the pilot study. First, due to logistical constraints, the allocation of classes to the intervention and control groups was carried out decentrally by teachers. While this might influence parameter estimates by affecting baseline balance between the study arms, we do not anticipate negative impacts on the other study objectives that are not contingent on unbiased effect estimates. For the effectiveness study, a blinded trial statistician will carry out the randomisation of classes to study arms using state-of-the-art techniques [68]. Second, the study is constrained by a short follow-up period of one month. While this follow-up is suitable for observing changes in pro-environmental intentions, it is presumably too short to observe changes in some secondary endpoints, including climate change-related self-efficacy or values and norms [69]. To examine both short- and medium-term effects of the PCS, we aim to conduct two follow-up surveys after 1 and 12 weeks in the future effectiveness study.

Third, the pilot study takes place amidst the COVID-19 pandemic, potentially affecting response rates due to, inter alia, non-attendance of students following mandatory quarantine measures or COVID-19 infections, thus limiting the generalisability of our findings. However, we anticipate that more students will attend schools during non-pandemic times, therefore rendering response rate estimations from the pilot study conservative. Forth, we acknowledge that no formal programme theory has been developed a priori to guide expectations how the PCS may be related to outcomes or interact with contextual factors. For programmes primarily developed by practitioners, however, recent guidance on the evaluation of complex interventions suggests that programme theory in these cases is often developed in parallel with the evaluation cycle [28]. We therefore plan to explore the applicability of the SIMPEA as programme theory for the PCS in a formative qualitative study comprising semi-structured interviews with students, teachers, school principals and members of the implementation team followed by transdisciplinary workshops. If suggested by interview and workshop results, we will develop the programme theory further by, inter alia, proposing outcomes not currently considered in the SIMPEA [36], mechanisms linking the PCS to outcomes and contextual factors at the level of students, teachers and schools that hinder or promote PCS implementation and potentially moderate intervention effectiveness. However, given the strong alignment of the SIMPEA with the objectives of the PCS, we anticipate that relatively minor adaptations to the original model will be necessary. Last, we acknowledge that different approaches may have been used at several points in designing this study. Given that the current understanding of issues explored in this study is limited and that our goal was to lay the groundwork for future work, we focused on foundational, yet methodologically important operationalisations of study processes, such as the proportion of students completing both baseline and follow-up surveys, providing a measure of participant retention, and the proportion of missing data and basic psychometric properties of scales, highlighting potential problems with data quality as well as floor and ceiling effects, respectively. We consider this pilot study as a learning opportunity for gauging the feasibility and potential implications of several methodological decisions made at this stage, potentially guiding the design and implementation of related research projects in Germany and internationally.

## Figures and Tables

**Table 1:** Primary and secondary outcomes.

Outcomes	Source of Scales and Adaptations	Response Options
**Primary outcome**		
Intentions to reduce one’s ecological footprint	Adaptation of the pro-environmental behaviour scales of Ojala (2012) [42,43] assessing intentions to reduce one’s ecological footprint during the upcoming seven days (five items).	six-point Likert scale
**Secondary outcomes**		
Intentions to enlarge one’s ecological handprint	Adaptation of the pro-environmental behaviour scales of Ojala (2012) [42,43] assessing intentions to enlarge one’s ecological handprint during the upcoming seven days (four items).	six-point Likert scale
Pro-environmental behaviours	Adaptation of the pro-environmental behaviour scales of Ojala (2012) [42,43]. Based on Ojala’s (2012) [43] distinction between *everyday behaviour* and *communication with others* we developed two new scales: one targeting *ecological footprint reduction behaviours* (five items) and the other targeting *ecological handprint enlargement behaviours* (four items). Behavioural options were selected that were specifically related to climate protection and that may have occurred with a certain probability in the last seven days. These items were supplemented by two statements about protest and political behaviour in the past 12 months.	six-point Likert scale
Climate change-related emotions	A list of 19 emotions based on terms for emotional reactions from de Moor et al. (2020) [14], Gagné and Krause (2021) [44] and Hickman et al. (2021) as well as from two reviews of Pihkala (2020) [45,46]. Feelings with positive connotations were added to the negative feelings surveyed in most studies of emotional responses toward climate change to mitigate a negative emotional bias.	five-point Likert scale (based on the Positive and Negative Affect Schedule (PANAS) [47].
Climate change-related risk perception	Based on the risk perception instrument by van der Linden (2015) [48] with the three areas *probability*, *severity*, and *current extent* of the consequences of global warming (two items each) with one *personal* and one *collective* reference point each. Items on the extent of concern were excluded to avoid redundancy (see below).	five-point Likert scale
Climate change-related concerns	Based on the measure of Schultz (2001) [49] used by Helm et al. (2018) [50] with the three dimensions *egoistic*, *altruistic* and *biospheric concerns*. For the egoistic and biospheric dimension, we selected one item each from the original scale, and for the altruistic dimension, we selected two items to distinguish between closer and more distant people.In addition, we survey the *tendency to deny the climate crisis* with four items based on Ojala’s (2012) [43] coping scale *de-emphasising the seriousness of climate change*.	six-point Likert scale
Climate change-related efficacy expectation	Following the work of Ojala (2013) [51], Hamann and Reese (2020) [52], Gardner and Neuber (2021) [53] and van Zomeren (2013) [54], we created one scale each for *self-efficacy expectations* (four items) and *collective efficacy expectations* (three items) related to action directed at the climate crisis. Following Hamann and Reese (2020) [52], we considered both scales to distinguish between *private* and *public efficacy*.	six-point Likert scale
Climate change-related values and norms	Two scales consisting of four items each to measure *altruistic values* and *biospheric values* [43]. A scale to assess *perceived environmental norms* was developed based on the biospheric values scale (four items) of Ojala (2012) [42]. Classmates were chosen as a reference group for the perceived norms.Three items on (post-)materialistic values [55] were used to cover the political dimension.	six-point Likert scale
Identification with civil engagement groups involved in climate action	We used the scale of Bamberg et al. (2015) [56] adapted by Wallis and Loy (2021) [40] to measure in-group identification with civil engagement groups involved in climate action (three items).	six-point Likert scale
Climate change-related knowledge	In the absence of previously validated scales, we developed a set of items on self-assessed climate change-related knowledge including environmentally sound products, climate change and possible actions for climate protection (three items).	six-point Likert scale

## Data Availability

Not applicable.

## References

[1] Solomon C.G., LaRocque R.C. (2019). Climate change—A health emergency. N. Engl. J. Med..

[2] Woodward A., Smith K.R., Campbell-Lendrum D., Chadee D.D., Honda Y., Liu Q., Olwoch J., Revich B., Sauerborn R., Chafe Z. (2014). Climate change and health: On the latest IPCC report. Lancet.

[3] Hathaway J., Maibach E.W. (2018). Health implications of climate change: A review of the literature about the perception of the public and health professionals. Curr. Environ. Health Rep..

[4] Clayton S., Manning C.M., College M., Speiser M., Hill A.N. (2017). Mental Health and Our Changing Climate: Impacts, Implications, and Guidance.

[5] Costello A., Abbas M., Allen A., Ball S., Bell S., Bellamy R., Friel S., Groce N., Johnson A., Kett M. (2009). Managing the health effects of climate change. Lancet.

[6] Masson-Delmotte V., Zhai P., Pörtner H.-O., Roberts D., Skea J., Shukla P.R., Pirani A., Moufouma-Okia W., Péan C., Pidcock R., IPCC (2018). Summary for policymakers. Global Warming of 1.5 °C. An IPCC Special Report on the Impacts of Global Warming of 1.5 °C above Pre-Industrial Levels and Related Global Greenhouse Gas Emission Pathways, in the Context of Strengthening the Global Response to the Threat of Climate Change, Sustainable Development, and Efforts to Eradicate Poverty.

[7] Masson-Delmotte V., Zhai P., Pirani A., Connors S.L., Péan C., Berger S., Caud N., Chen Y., Goldfarb L., Gomis M.I., IPCC (2021). Summary for policymakers. Climate Change 2021: The Physical Science Basis. Contribution of Working Group I to the Sixth Assessment Report of the Intergovernmental Panel on Climate Change.

[8] McMichael A.J., Campbell-Lendrum D., Kovats S., Edwards S., Wilkinson P., Wilson T., Nicholls R., Hales S., Tanser F., Le Sueur D., Ezzati M., Lopez A.D., Rodgers A.A., Murray C.J.L. (2004). Global climate change. Comparative Quantification of Health Risks: Global and Regional Burden of Disease Due to Selected Major Risk Factors.

[9] Sanson A.V., Van Hoorn J., Burke S.E.L. (2019). Responding to the impacts of the climate crisis on children and youth. Child Dev. Perspect..

[10] Burke S.E.L., Sanson A.V., Van Hoorn J. (2018). The psychological effects of climate change on children. Curr. Psychiatry Rep..

[11] Van Susteren L., Al-Delaimy W.K., Al-Delaimy W.K., Ramanathan V., Sánchez Sorondo M. (2020). Psychological impacts of climate change and recommendations. Health of People, Health of Planet and Our Responsibility.

[12] Clayton S. (2020). Climate anxiety: Psychological responses to climate change. J. Anxiety Disord..

[13] Hickman C., Marks E., Pihkala P., Clayton S., Lewandowski R.E., Mayall E.E., Wray B., Mellor C., van Susteren L. (2021). Climate anxiety in children and young people and their beliefs about government responses to climate change: A global survey. Lancet Planet. Health.

[14] De Moor J., Uba K., Wahlström M., Wennerhag M., Vydt M.D. (2020). Protest for a Future II: Composition, Mobilization and Motives of the Participants in Fridays for Future Climate Protests on 20–27 September, 2019, in 19 Cities around the World. https://www.diva-portal.org/smash/get/diva2:1397070/FULLTEXT01.pdf.

[15] Treude K.D. (2021). Environmental Action in Germaringen-Young People Plant Trees for Climate Protection. Allgäuer Ztg. https://www.allgaeuer-zeitung.de/allgaeu/kaufbeuren/klimaschutz-im-allgaeu-jugendliche-pflanzen-baeume-in-germaring_arid-343120.

[16] Kasser T. (2017). Living both well and sustainably: A review of the literature, with some reflections on future research, interventions and policy. Philos. Trans. R. Soc. Lond. Ser. A Math. Phys. Eng. Sci..

[17] Grund J., Brock A. (2019). Why we should empty Pandora’s box to create a sustainable future: Hope, sustainability and its impli-cations for education. Sustainability.

[18] Keller L., Stötter J., Oberrauch A., Kuthe A., Körfgen A., Hüfner K. (2019). Changing climate change education: Exploring moderate constructivist and trans-disciplinary approaches through the research-education co-operation k.i.d.Z.21. GAIA-Ecol. Perspect. Sci. Soc..

[19] Grund J., Brock A. (2020). Education for Sustainable Development in Germany: Not Just Desired but Also Effective for Transformative Action. Sustainability.

[20] Holst J., Brock A., Singer-Brodowski M., De Haan G. (2020). Monitoring Progress of Change: Implementation of Education for Sustainable Development (ESD) within Documents of the German Education System. Sustainability.

[21] UNESCO (2021). Learn for Our Planet: A Global Review of How Environmental Issues Are Integrated in Education. https://unesdoc.unesco.org/ark:/48223/pf0000377362.

[22] Boeve-de Pauw J., Gericke N., Olsson D., Berglund T. (2015). The effectiveness of education for sustainable development. Sustainability.

[23] Boeve-de Pauw J., Van Petegem P. (2011). The effect of flemish eco-schools on student environmental knowledge, attitudes, and affect. Int. J. Sci. Educ..

[24] Boeve-de Pauw J., Van Petegem P. (2013). The effect of eco-schools on children’s environmental values and behaviour. J. Biol. Educ..

[25] Olsson D., Gericke N., Pauw J.B.-D., Berglund T., Chang T. (2019). Green schools in Taiwan–Effects on student sustainability consciousness. Glob. Environ. Chang..

[26] UNESCO (2017). Education for Sustainable Development Goals: Learning Objectives. https://www.unesco.de/sites/default/files/2018-08/unesco_education_for_sustainable_development_goals.pdf.

[27] UNESCO (2012). Education for Sustainable Development: Building a Better, Fairer World for the 21st Century. https://unesdoc.unesco.org/ark:/48223/pf0000216673.

[28] Skivington K., Matthews L., Simpson S., Craig P., Baird J., Blazeby J., Boyd K., Craig N., French D., McIntosh E. (2021). A new framework for developing and evaluating complex interventions: Update of Medical Research Council guidance. BMJ.

[29] Bundesministerium für Bildung und Forschung (2021). What is Education for Sustainable Development?. https://www.bne-portal.de/bne/de/einstieg/was-ist-bne/was-ist-bne_node.html;jsessionid=BAF2E8BCBFE90DAF2C33E22B3CEFB6D5.live381.

[30] Cardinale B.J., Duffy J.E., Gonzalez A., Hooper D.U., Perrings C., Venail P., Narwani A., Mace G.M., Tilman D., Wardle D.A. (2012). Biodiversity loss and its impact on humanity. Nature.

[31] Schlosberg D., Collins L.B. (2014). From environmental to climate justice: Climate change and the discourse of environmental justice. WIREs Clim. Chang..

[32] Dietz S., Stern N. (2009). On the Timing of Greenhouse Gas Emissions Reductions: A Final Rejoinder to the Symposium on “The Economics of Climate Change: The Stern Review and its Critics”. Rev. Environ. Econ. Policy.

[33] McPhearson T., Iwaniec D.M., Bai X. (2016). Positive visions for guiding urban transformations toward sustainable futures. Curr. Opin. Environ. Sustain..

[34] Gabrys J., Yusoff K. (2012). Arts, Sciences and Climate Change: Practices and Politics at the Threshold. Sci. Cult..

[35] Ogilvie D., Foster C.E., Rothnie H., Cavill N., Hamilton V., Fitzsimons C.F., Mutrie N. (2007). Interventions to promote walking: Systematic review. BMJ.

[36] Fritsche I., Barth M., Jugert P., Masson T., Reese G. (2018). A Social Identity Model of Pro-Environmental Action (SIMPEA). Psychol. Rev..

[37] Ajzen I., Fishbein M., Albarracín D., Johnson B.T., Zanna M.P. (2005). The influence of attitudes on behavior. The Handbook of Attitudes.

[38] Klöckner C.A. (2013). A comprehensive model of the psychology of environmental behaviour—A meta-analysis. Glob. Environ. Chang..

[39] Schwartz S.H., Howard J.A., Rushton J.P., Sorrentino R.M. (1981). A normative decision-making model of altruism. Altruism and Helping Behavior.

[40] Wallis H., Loy L.S. (2021). What drives pro-environmental activism of young people? A survey study on the Fridays for Future movement. J. Environ. Psychol..

[41] Mackay C.M., Schmitt M.T., Lutz A.E., Mendel J. (2021). Recent developments in the social identity approach to the psychology of climate change. Curr. Opin. Psychol..

[42] Ojala M. (2012). Hope and climate change: The importance of hope for environmental engagement among young people. Environ. Educ. Res..

[43] Ojala M. (2012). How do children cope with global climate change? Coping strategies, engagement, and well-being. J. Environ. Psychol..

[44] Gagné J., Krause L.-K. (2021). Unifying or Divisive? Climate Action and Social Cohesion in Germany. https://www.moreincommon.de/media/13ip5esl/more_in_common_studie_klima_zusammenhalt.pdf.

[45] Pihkala P. (2020). Anxiety and the ecological crisis: An analysis of eco-anxiety and climate anxiety. Sustainability.

[46] Pihkala P. (2020). Eco-Anxiety and Environmental Education. Sustainability.

[47] Breyer B., Bluemke M. (2016). German Version of the Positive and Negative Affect Schedule PANAS. http://zis.gesis.org/DoiId/zis242.

[48] Van der Linden S. (2015). The social-psychological determinants of climate change risk perceptions: Towards a comprehensive model. J. Environ. Psychol..

[49] Wesley Schultz P. (2001). The structure of environmental concern: Concern for self, other people, and the biosphere. J. Environ. Psychol..

[50] Helm S.V., Pollitt A., Barnett M.A., Curran M.A., Craig Z.R. (2018). Differentiating environmental concern in the context of psychological adaption to climate change. Glob. Environ. Chang..

[51] Ojala M. (2013). Coping with climate change among adolescents: Implications for subjective well-being and environmental engagement. Sustainability.

[52] Hamann K.R.S., Reese G. (2020). My Influence on the World (of Others): Goal Efficacy Beliefs and Efficacy Affect Predict Private, Public, and Activist Pro-environmental Behavior. J. Soc. Issues.

[53] Gardner B.G., Neuber M. (2021). Fighting Every Crisis in the Wake of COVID-19: Shifting Grounds for Mobilization among Fridays for Future Protesters in Germany. https://osf.io/rsy92.

[54] Van Zomeren M. (2013). Four Core Social-Psychological Motivations to Undertake Collective Action: Four motivations for collective action. Soc. Personal. Psychol. Compass.

[55] van Stekelenburg J., Walgrave S., Klandermans B., Verhulst J. (2012). Contextualizing contestation. Framework, design and data. Mobilization.

[56] Bamberg S., Rees J., Seebauer S. (2015). Collective climate action: Determinants of participation intention in community-based pro-environmental initiatives. J. Environ. Psychol..

[57] Hoebel J., Müters S., Kuntz B., Lange C., Lampert T. (2015). Measuring subjective social status in health research with the German version of the MacArthur Scale. Bundesgesundheitsblatt.

[58] Eldridge S.M., Costelloe C.E., Kahan B.C., Lancaster G.A., Kerry S.M. (2016). How big should the pilot study for my cluster randomised trial be?. Stat. Methods Med. Res..

[59] Graham J.W. (2009). Missing Data Analysis: Making It Work in the Real World. Annu. Rev. Psychol..

[60] Tabachnick B.G., Fidell L.S. (2019). Using Multivariate Statistics.

[61] Chan A.-W., Tetzlaff J.M., Altman D.G., Laupacis A., Gøtzsche P.C., Krleža-Jerić K., Hróbjartsson A., Mann H., Dickersin K., Berlin J.A. (2013). SPIRIT 2013 Statement: Defining Standard Protocol Items for Clinical Trials. Ann. Intern. Med..

[62] Eldridge S.M., Chan C.L., Campbell M.J., Bond C.M., Hopewell S., Thabane L., Lancaster G.A. (2016). CONSORT 2010 statement: Extension to randomised pilot and feasibility trials. BMJ.

[63] Thabane L., Lancaster G. (2019). A guide to the reporting of protocols of pilot and feasibility trials. Pilot Feasibility Stud..

[64] Autorengruppe Bildungsberichterstattung (2020). Education in Germany 2020—An Indicator-Based Report with an Analysis of Education in a Digitised World.

[65] Neuber M., Gardner B.G., De Moor J., Uba K., Wahlström M., Wennerhag M., De Vydt M. (2020). Germany. Protest for a Future II: Composition, Mobilization and Motives of the Participants in Fridays for Future Climate Protests on 20–27 September, 2019, in 19 Cities around the World.

[66] Neuber M., Kocyba P., Gardner B.G., Haunss S., Sommer M. (2020). The same, only different: Fridays for Future protesters in European comparison. Fridays for Future-Youth against Climate Change. Contours of the International Protest Movement.

[67] Eldridge S., Kerry S. (2012). A Practical Guide to Cluster Randomised Trials in Health Services Research.

[68] Ivers N.M., Halperin I.J., Barnsley J., Grimshaw J.M., Shah B.R., Tu K., Upshur R., Zwarenstein M. (2012). Allocation techniques for balance at baseline in cluster randomized trials: A methodological review. Trials.

[69] Bandura A. (1997). Self-Efficacy: The Exercise of Control.

